# Immunotherapy: A Novel Era of Promising Treatments for Multiple Myeloma

**DOI:** 10.3390/ijms19113613

**Published:** 2018-11-15

**Authors:** Maria Castella, Carlos Fernández de Larrea, Beatriz Martín-Antonio

**Affiliations:** 1 Department of Hematology, Hospital Clinic, IDIBAPS, 08036 Barcelona, Spain; mcastella@clinic.cat (M.C.); cfernan1@clinic.cat (C.F.d.L.); 2 Josep Carreras Leukaemia Research Institute, 08036 Barcelona, Spain

**Keywords:** multiple myeloma, monoclonal antibodies, antibodies drug conjugates, immunocheckpoint inhibitors, chimeric antigen receptor (CAR)-modified T cells

## Abstract

Multiple myeloma (MM) remains an incurable hematological malignancy characterized by clonal proliferation of malignant plasma cells in bone marrow. In the last 20 years, the introduction of autologous stem cell transplantation, followed by proteasome inhibitors and immunomodulatory agents, increased the survival of MM patients by 50%. However, still a high proportion of patients relapse and become refractory, especially, high-risk patients with adverse cytogenetics where these treatment combinations have shown limited benefit. Therefore, novel strategies, such as immunotherapy, have been developed in the last few years to help improve the survival of these patients. Immunotherapy treatments include a high number of different strategies used to attack the tumor cells by using the immune system. Here, we will review the most successful immunotherapy strategies published up to date in patients with relapsed or refractory (R/R) MM, including monoclonal antibodies targeting specific antigens on the tumor cells, antibodies combined with cytotoxic drugs or Antibodies Drug Conjugates, immune checkpoint inhibitors which eliminate the barriers that damper immune cells and prevent them from attacking tumor cells, bi-specific T-cell engagers antibodies (BiTEs), bi-specific antibodies and the infusion of chimeric antigen receptor-modified T cells. We overview the results of clinical studies that have been presented up to date and also review pre-clinical studies describing potential novel treatments for MM.

## 1. Multiple Myeloma, an Incurable Disease with Current Treatments

Multiple myeloma (MM) remains an incurable hematologic malignancy responsible for 1% of all cancers and 15–20% of all hematological malignancies. The incidence in Europe is 4.5–6.0/100,000/year with a median age at diagnosis around 70 years, a mortality rate of 4.1/100,000/year [1,2] and a 5 years’ survival rate of 50.7% [3]. Unfortunately, over the past decade, the rates for new MM cases have increased an average of 0.8% per year [3]. MM is characterized by clonal expansion of malignant plasma cells in the bone marrow, which leads to an excessive production of monoclonal immunoglobulin (Ig) detectable in serum and/or urine. Associated clinical symptoms include osteolytic lesions, anemia, renal dysfunction, hypercalcemia, infections and other related organ dysfunction [4].

### Evolution of MM Treatment

MM treatment has undergone a gradual series of changes over the last decades that have improved the survival of patients. However, the prognosis for MM patients is still poor in terms of curability and lifespan under treatment when compared to other hematological malignancies, such as Hodgkin lymphoma or chronic myeloid leukemia. Nowadays, a high variety of treatments are being evaluated in clinical trials to continue improving the survival of patients. Back in 1962, initial regimens based on a melphalan-prednisone achieved very low rates of complete remission. This was significantly improved with the incorporation of autologous stem cell transplantation (ASCT) in 1996. Afterwards, the introduction of proteasome inhibitors (PI) in 2003 [5], as well as the new generation of immunomodulatory drugs (IMiDs) in 2005, achieved an increased overall survival (OS) from 14.8 to 30.9 months [6]. Importantly, treatments based on the combination of these drugs have shown an improved survival of 50% (44.8 vs. 29.9 months) when comparing before and after 2001 [6]. Currently, the first line of treatment for newly diagnosed MM patients consists of an induction treatment combining one PI, one IMiD and corticosteroids (dexamethasone). This induction treatment is followed by ASCT and a maintenance phase, usually based on lenalidomide. Consolidation chemotherapy regimen after ASCT, usually similar to the induction regimen, is under investigation. With these protocols, an extended survival from three to eight years was achieved [4,7]. Using bortezomib/lenalidomide/dexamethasone (VRD) combination followed by ASCT and 1-year lenalidomide maintenance, 58% of very good partial responses (VGPR) are achieved after induction, and after ASCT and consolidation therapy, VGPR or better achieve 70% and 87%, respectively [8]. However, these PI-based regimens are not always effective, as 19% of patients do not respond to PI when used as induction treatment, and only 50% of patients with relapsed MM respond to this therapy thus becoming utterly refractory [9,10]. In fact, the natural history of MM is relapse until refractory disease without reaching a plateau of survival. Thus, less than 10% of patients achieve sustained complete responses (CR) beyond 5–10 years after ASCT. Moreover, relapsed or refractory (R/R) MM patients to at least 3 lines of treatment with PIs and IMiDs present only a median OS of 8 months [11]. Therefore, there is a clear unmet medical need in patients with R/R MM. Novel strategies are required to improve their survival, especially in high-risk patients with adverse cytogenetics where these combinations have shown limited benefit [12,13].

In this regard, as a result of a deeper understanding of the plasma cell biology, novel treatments are currently being tested in this group of patients. Recent developed drugs include the next generation of PIs (carfilzomib, ixazomib, marizomib, and oprozomib), small and targeted molecules such as histone deacetylase inhibitors, venetoclax, selinexor, Hsp90 inhibitors and PI3K/AKT/mTOR inhibitors which are currently under development. Interestingly, immunotherapy has arisen as a new modality treatment with very promising results and less toxic effects. However, side effects associated to this treatment modality, such as autoimmunity and cytokine release syndrome (CRS) still need to be ameliorated. Immunotherapy includes a high variety of treatments, such as monoclonal antibodies, bi-specific T cell engaging antibodies (BiTEs), bi-specific antibodies, antibody-drug conjugates (ADC), immune checkpoint inhibitors and adoptive cell immunotherapy. In this review, we will present the main immunotherapy strategies currently being used for R/R MM and other promising treatments currently being developed at a pre-clinical stage that may constitute the future of standards of clinical care for patients with MM. The most relevant studies are summarized in Table 1.

## 2. Main Immunotherapy Strategies Currently Being Used or Tested for Relapsed/Refractory MM Patients

### 2.1. Monoclonal Antibodies Targeting Antigens Expressed on MM Cells

A successful and non-toxic immunotherapy treatment based on antibodies that target antigens expressed on tumor cells requires high specific and restricted expression of the target antigen. Whereas normal plasma cells express CD38, CD138, CD19 and CD45, malignant PCs loose CD45 and CD19 and usually acquire high expression of CD56 and CD117 [14,15]. At the “Ninth International Workshop on Leukocyte Antigens” a panel of surface plasma cell markers expressed in newly diagnosed MM patients was presented. This panel includes CD150 (SLAMF1), CD48 (SLAMF2), CD229 (SLAMF3), CD352 (SLAMF6), CD319 (SLAMF7 or CS1), CD272, CD86, CD200, and CD184. A later study comparing these markers in plasma cells from newly diagnosed patients, R/R MM, plasma cell leukemia patients and healthy individuals concluded that SLAMF2, SLAMF3, SLAMF7 and CD272 could be other possible targets for immunotherapy [16]. Currently, only Daratumumab (anti-CD38), and Elotuzumab (anti-CS1) have received approval by the Food and Drug Administration (FDA) and European Medicines Agency (EMA) for their use in patients with MM.

Daratumumab (Darzalex) is a monoclonal antibody that recognizes CD38 and induces tumor cell death not only through complement-dependent cytotoxicity (CDC) and antibody-dependent cell-mediated cytotoxicity (ADCC) [71] but also through antibody-mediated cellular phagocytosis [72]. In 2016, the FDA approved daratumumab as monotherapy for the treatment of R/R MM patients who received ≥3 prior treatments that included a PI and an IMiD or those who were double refractory to a PI and an IMiD [73]. This approval was based on initial results of the GEN501 [17] and SIRIUS studies [18] showing OR of 36% and 29% after a median follow-up of 16.9 months and 9.3 months, respectively. Importantly a deepening of response over time that included CRs and stringent CR (sCRs) was also observed. Afterwards, an updated analysis of these patients, showed OR of 31.1%, and a median duration of response of 7.6 months, with a progression free survival (PFS) and OS of 4 and 20.1 months, respectively [19]. Daratumumab has also been tested in combination with other agents. Combination of daratumumab with bortezomib and dexamethasone has shown longer PFS than bortezomib and dexamethasone alone, with OR of 82.9% vs. 63.2%, and CR or better of 19.2% vs. 9%. At a median follow-up of 7.4 months, the median PFS was not reached in the daratumumab group, being 7.2 months in the control group. However, the combination of daratumumab with other drugs was also associated with some grade 3 and 4 adverse events [20]. An updated analysis of this study showed that at a median of 19.4 months, daratumumab plus bortezomib and dexamethasone prolonged PFS (16.7 vs. 7.1 months) and improved the OR (83.8% vs. 63.2%) [21]. Daratumumab has also been tested in combination with lenalidomide and dexamethasone. A phase III study with 569 R/R MM patients showed that daratumumab improved PFS at 12 months (83.2% vs. 60.1%), OR (92.9% vs. 76.4%), and higher CR or better (43.1% vs. 19.2%) were obtained. Unfortunately, again, daratumumab associated with higher grade 3 and 4 adverse events including neutropenia (51.9% vs. 37.0%) and higher rate of infection (28.3 vs. 22.8%) [22]. An updated analysis of this study at 25.4 months showed that daratumumab plus lenalidomide/dexamethasone vs. lenalidomide/dexamethasone alone prolonged PFS (median not reached vs. 17.5 months). The OR was 92.9% vs. 76.4% and CR or better was 51.2% vs. 21.0%. PFS was significantly prolonged for daratumumab group, a benefit also maintained in high-risk patients (PFS of 22.6 vs. 10.2 months) [23]. Finally, among all treatment combinations in R/R MM, a recent meta-analysis of 24 randomized controlled trials performed in published studies up to June 2017 showed that the combination of daratumumab, lenalidomide, and dexamethasone achieved better efficacy than other regimens in terms of time to progression and PFS [74]. Despite these positive results, loss of expression of the target antigen (CD38) remains as an important problem associated to daratumumab treatments, as tumor cells stop responding to the therapy. Different mechanisms have been suggested to be responsible for antigen loss, such as uptake of CD38 by monocytes or granulocytes through trogocytosis [75] or through release in microvesicles [76]. On the other side, loss of CD38 has been described to decrease the adenosine effect and create a less immunosuppressive environment [77], which could facilitate further therapies. Additional studies are required to elucidate the consequences of loss of target antigen and to continue improving clinical results.

Elotuzumab (EMPLICITI) is a humanized monoclonal antibody which recognizes SLAMF7 or CS1. SLAM family of receptors are type I transmembrane glycoproteins belonging to the Ig superfamily, which are adhesion molecules expressed on different hematopoietic cells [78]. CS1 was described to be highly expressed on MM cells and normal plasma cells. Some other hematological cells, such as Natural Killer (NK) cells, some T cell subsets and dendritic cells, also show low levels of CS1 expression [79]. Interestingly, elotuzumab has shown little or no direct cytotoxic activity on MM cells in vitro. However, CS1 is a positive regulator of NK cell activation [80]. In this way, elotuzumab anti-MM activity has been associated to activation of ADCC mediated by *NK cells* and also to enhanced NK cytotoxicity independent of ADCC [81,82]. Moreover, elotuzumab promotes antibody-dependent cellular phagocytosis of macrophages contributing to the elotuzumab antitumor potency [83].

In patients with R/R MM, elotuzumab has not shown objective responses as a single agent, achieving only 26.5% of stable disease at the highest dose tested and showing mild to moderate adverse events [24]. However, a higher efficacy was obtained in combination regimens. In a phase I study including 29 patients with R/R MM, elotuzumab with lenalidomide and dexamethasone showed 82% of OR and median time to progression was not reached for patients treated with elotuzumab after a median of 16.4 months [25]. A subsequent phase 3 study (ELOQUENT-2 study) comparing elotuzumab with lenalidomide and dexamethasone vs. lenalidomide and dexamethasone, showed that after a median of 24.5 months, PFS at 2 years was 41% for elotuzumab vs. 27% in the control group. Median PFS was 19.4 vs. 14.9 months in the control group and OR was 79% vs. 66% in the control group [26]. These results led to the FDA approval of elotuzumab for use in combination with lenalidomide and dexamethasone for treatment of R/R MM patients, in November 2015 [84,85]. An updated analysis at 3 and 4 years of the ELOQUENT-2 study concluded that risk of disease progression/death was reduced by 27% in the Elotuzumab arm, and OS and PFS demonstrated a trend in favour of elotuzumab, showing at 1, 2, 3 and 4-years an OS of: 91% vs. 83%, 73% vs. 69%, 60% vs. 53% and 50% vs. 43%. PFS at 1, 2, 3 and 4-year was of: 69% vs. 57%, 41% vs. 28%, 27% vs. 19% and 21% vs. 14%. Adverse events were similar in both groups [27,28]. Moreover, high-risk patients had a 36% reduction in the risk of progression/death when treated with Elotuzumab.

B cell maturation antigen (BCMA) is the tumor necrosis factor superfamily member 17, a transmembrane glycoprotein involved in the regulation of B cell maturation and survival [86,87]. BCMA is an ideal target for MM due to its specific and restricted expression in MM cells. The main success of BCMA in immunotherapy has occurred in the field of chimeric antigen receptor (CAR) T cell therapy, as discussed later. However, in the field of Antibody Drug Conjugates (ADC), three compounds are being tested: GSK2857916, HDP-1 and MEDI2228. GSK2857916 is a humanized afucosylated anti-BCMA antibody coupled to maleimidocaproy and monomethyl auristatin, which has shown potent anti-MM activity, in part by triggering ADCC and antibody-dependent cellular-mediated phagocytosis. Its effect is enhanced with lenalidomide [88]. When testing GSK2857916 as monotherapy for R/R MM patients in a phase I study, it obtained 60% of OR with a median PFS of 7.9 months [43]. HDP-1 is another anti-BCMA antibody with a payload of maleimide-amanitin which has demonstrated potent anti-MM activity in vitro and in murine and Cynomolgus monkeys models [44]. Finally, MEDI2228 is an ADC composed of a fully human anti-BCMA antibody conjugated to a pyrrolobenzodiazepine dimer with potent in vitro and in vivo anti-MM activity in murine models [45].

Other monoclonal antibodies have been tested in patients with R/R MM with limited success in initial studies. These include antibodies against IL6, CD56, CD138 and CD40. IL6 enhances the survival of MM cells and limits the benefits of other MM treatments [89]. Therefore, siltuximab (CNTO 328), a monoclonal antibody against IL6 was analyzed in a Phase II study. Siltuximab alone did not show any benefit and very limited benefit was also obtained when in combination with dexamethasone [29]. A lack of benefit of this novel drug was confirmed in an additional Phase II study that analyzed siltuximab in combination with bortezomib, melphalan and prednisone [30].

Lorvotuzumab is a humanized monoclonal antibody against CD56. CD56 is highly expressed in malignant plasma cells [90] but also in other immune cells, such as NK cells. Lorvotumumab as a single agent showed limited response [46]. Further studies analyzed Lorvotumumab as an ADC combining it with maytansine (named Lorvotuzumab mertansine). This ADC was tested in combination with lenalidomide and dexamethasone in a Phase II study in 2014. Limited responses were obtained with OR of 56.4%. Neurotoxicity was detected as the most common adverse event [47]. Due to its reduced benefit no further studies have been conducted.

CD138 is a member of the Syndecan family with a role in cell-cell contacts. It participates in the regulation of various processes such as cell proliferation, apoptosis, metastasis and angiogenesis. It is highly expressed in MM cells, but also in epithelial tissues (reviewed in [91]). A murine/human chimeric antibody against CD138 was tested as an ADC, by conjugating the antibody to the maytansinoid drug DM4 (named Indatuximab ravtansine or BT062). BT062 has shown anti-MM activity in vitro and in murine models [92]. However, as a single-agent in MM patients, BT062 did not achieve responses [48]. In combination with lenalidomide and lenalidomide/dexamethasone an additive or synergistic anti-MM activity was shown in murine models [49]. CD138 has also been used in pre-clinical models of radioimmunotherapy, an approach which couples an antibody with a radionuclide that has a cytotoxic effect. This combination was shown to delay disease progression in xenograft MM murine models [93].

Lucatumumab and dacetuzumab are two monoclonal antibodies against CD40 which have been tested in patients with R/R MM. Lucatumumab is a human monoclonal antibody that was evaluated in a phase 1 study in 28 R/R MM patients. The therapy was being well tolerated. However, only one patient (4%) maintained a PR for ≥8 months [31]. Dacetuzumab is a humanized monoclonal antibody which was tested in 44 patients, and only 20% of patients achieved stable disease [32]. Dacetuzumab was also tested in combination with lenalidomide and dexamethasone and only limited responses were obtained with 39% of OR [33]. No further studies have been published in MM patients with these antibodies.

IPH2101 is a human monoclonal antibody against inhibitory killer-cell immunoglobulin-like receptors (KIRs) on NK cells that inhibit the cytotoxic activity of NK cells [94]. By blocking these inhibitory signals, NK cells should be activated against MM cells. Unfortunately, phase I studies in R/R MM patients using IPH2101, both as a single agent or in combination with lenalidomide, failed to obtain responses. As a single agent, IPH2101 was well tolerated and no OR were detected [34]. In combination with lenalidomide, in 15 patients, IPH2101 achieved only 33.3% of OR with a median PFS of 24 months [35].

Cetuximab, a chimeric human-murine antibody against EGFR was also tested in a Phase II study in R/R MM patients, as single agent and in combination with dexamethasone. However, no ORs were detected in combination with dexamethasone. 47% of patients achieved stable disease [36].

### 2.2. Bi-Specific T-Cell Engagers Antibodies (BiTEs) and Bi-Specific Antibodies for R/R MM Patients

BiTEs have shown promising results in the treatment of R/R MM in pre-clinical studies. BiTEs are designed to bind a tumor cell and an immune cell by engaging usually CD3 with an antigen expressed in the tumor cell. Consequently, the T cell becomes activated and attacks the target cell [95]. There are different BiTEs targeting BCMA and CD3 (BI 836909, EM801 and JNJ-64007957), which have shown potent anti-MM activity in vitro and in vivo murine and monkey models of MM [50,51,52]. These BiTEs and also PF-06863135 are currently being tested in clinical trials in patients with MM (NCT02514239, NCT03145181, NCT03269136 and NCT03269136). Other promising BiTEs, which are at a pre-clinical stage, include one combining CD138 and CD3, which has shown potent anti-MM activity in vitro and in murine models [53].

Also bi-specific antibodies are being tested for the treatment of MM. NKG2D is an activating receptor expressed on NK cells, CD8^+^ T cells, γδ T cells and NKT cells. Simultaneous targeting of NKG2D and CS1 should engage both innate and adaptive immune cells to target MM cells. A bi-specific antibody combining NKG2D with CS1 has shown in vitro and in vivo anti-MM activity [54]. BiFabs are bi-specific antibodies which target two different epitopes on the same protein. BiFabs against BCMA and CS1 have been tested and compared, demonstrating that BiFab-BCMA had higher anti-MM activity than BiFab-CS1. Moreover, BiFab-BCMA anti-MM activity was comparable to that of CART-BCMA cells in vitro and in vivo [55].

### 2.3. Monoclonal Antibodies Targeting Immune Checkpoints between Immune and MM Cells

Immune checkpoints are negative signals established between immune and tumor cells that hamper immune cells and prevent them from eliminating malignant cells. Allison J. pioneered studies blocking immune checkpoints and demonstrated enhanced anti-tumor responses. These studies led to the development of very successful cancer therapies, especially in solid tumors [96,97]. Antibodies such as ipilimumab, which blocks CTLA-4, pembrolizumab and nivolumab, which block PD-1 in T cells, and atezolizumab, durvalumab and avelumab, which block PD-L1 on tumor cells, have been developed and approved by the FDA for solid tumors [98,99,100,101,102]. However, targeting immune checkpoints has not been very successful in MM as of yet.

Pre-clinical studies showed that immune and MM cells from patients present higher expression of PD-1 and PD-L1 than healthy individuals, and that the blockade of PD-1/PD-L1 abrogates MM growth, which is further enhanced with lenalidomide combination [103]. Moreover, in murine MM models, low dose irradiation, as lymphodepletive chemotherapy, plus PD-L1 blockade provided synergistic anti-tumor efficacy [104]. However, when these inhibitors were tested in patients with MM, results were disappointing. As single agents, PD-1 inhibitors were not successful. Whereas pembrolizumab has not been tested as a single agent in R/R MM patients, nivolumab was tested in 27 R/R MM patients (NCT01592370). OR occurred only in one patient (4%) and moreover, adverse events occurred in 52% with 19% of them being serious adverse events [37]. Afterwards, combination studies with IMiDs showed higher efficacy but also higher toxicity. Pembrolizumab combined with pomalidomide and dexamethasone in a Phase II trial with 48 R/R MM patients showed severe adverse events in 40% of patients. ORs were of 60%, including 8% of stringent CR/CR, 19% VGPR and 33% PR. At median follow-up of 15.6 months, PFS was 17.4 months and OS was not reached [38]. Pembrolizumab was also combined with lenalidomide and low-dose dexamethasone in a phase I study including 51 patients. 65% of patients experienced high grade adverse events. Only 40 patients continued in the study, obtaining 50% of OR and a median duration of response of 11.3 months [39]. However, later studies in a phase III trial comparing pembrolizumab, pomalidomide and low dose dexamethasone vs. pomalidomide and dexamethasone (NCT02576977) [105], and a second study in newly diagnosed MM patients comparing pembrolizumab with lenalidomide and low dose dexamethasone vs. lenalidomide and low dose dexamethasone (NCT02579863) [106] reported a high number of deaths related to the high toxicity of the treatment. Non-disease progression causes of death identified in the pembrolizumab arm included intestinal ischemia, pulmonary embolism, pneumonia, sudden death, large intestine perforation, myocarditis, Stevens-Johnson syndrome, myocardial infarction, pericardial hemorrhage, cardiac failure, cardio-respiratory arrest, respiratory tract infection, neutropenic sepsis, sepsis, multiple organ dysfunction and respiratory failure. Consequently, both studies were halted in 2017 [40]. 

Nivolumab, another PD-1 inhibitor, is currently being used in combination with different IMiDs at several clinical trials (NCT02726581, NCT02612779, NCT03333746, NCT03023527, NCT03605719, NCT03184194, NCT01592370). However, clinical results are not available yet. Additional information regarding these clinical trials is summarized in Table 1. In addition, PD-L1 inhibitors (durvalumab and atezolizumab) are also being assessed in R/R MM patients in different clinical trials. Durvalumab is being evaluated in combination with different IMiDs, dexamethasone and daratumumab (NCT02616640, NCT02807454, NCT03000452); and atezolizumab with cobimetinib and venetoclax (NCT03312530), and with different combinations of IMiDs and daratumumab (NCT02431208). Additional information is summarized in Table 1.

### 2.4. Chimeric Antigen Receptor (CAR)-T Cell Immunotherapy

Genetically modifying autologous T cells to express chimeric antigen receptors (CARs) thus redirecting them to eliminate tumor cells or other harmful cells is a new and revolutionary therapeutic modality for cancer treatment [62,107,108,109,110,111,112,113,114]. CARs are composed of an extracellular region responsible for binding to a particular antigen and an intracellular region that promotes T cell cytotoxic activity and proliferation. CAR binding to the selected antigen is usually mediated by a single chain variable fragment (scFv) of a monoclonal antibody and is MHC-independent. This scFv is combined with an intracellular co-stimulatory domain (usually CD28 or 4-1BB) and a pro-activator cytotoxic domain (CD3ζ) [115,116,117].

Anti-CD19 CAR is a paradigm for CAR T cell therapy. It has shown impressive response rates in CD19^+^ R/R hematological malignancies, especially in B-cell acute lymphoblastic leukemia (B-ALL), non-Hodgkin’s lymphoma (NHL), and chronic lymphocytic leukemia (CLL). Response rates range from 50–85% depending on the type of B-cell malignancy and CAR construct, with quite remarkable DFS and OS. In terms of safety, patients who respond to anti-CD19 therapy usually develop persistent B-cell aplasia, which is then treated with gamma globulins as a replacement therapy, and transitory cytokine release syndrome (CRS), manageable in the vast majority of cases. However, some patients experience severe CRS with hypotension, pulmonary edema, coagulopathy, vascular leak and neurotoxicity [114,117,118,119,120]. This success led to the rapid development of CAR T cell field. The FDA and European Medicines Agency (EMA) have already approved two CAR19 products (tisagenlecleucel and axicabtagene ciloleucel) for the treatment of B-ALL and large B-cell lymphoma (DLBCL). Other CARs for hematologic and solid tumors are currently being developed at the pre-clinical or clinical stage. The success of new CAR T cell therapies relies on selecting the right antigen to target. The antigen should be expressed broadly in tumors of a given type, but should not be present in healthy tissues, as to avoid off-target effects and associated toxicity. Therefore, the number of antigens to target is limited. As we describe below, new approaches include dual targeting, targeting cancer neoantigens or cancer-specific protein conformations expressed on the surface of cancer cells. Here, we describe the present and future of CAR T cell therapies for MM. There are currently 32, completed or on-going, clinical trials involving CAR T cell therapy in MM. The vast majority of them are evaluating the efficacy of anti-BCMA CAR and a few assessing anti-CD19 or anti-CD138 CAR.

#### 2.4.1. CAR BCMA

As previously mentioned, BCMA constitutes an ideal candidate for targeted MM therapies, as its expression is restricted to plasma cells, being the expression in MM cells higher than in normal PCs [121,122]. Initial studies using CAR BCMA in R/R MM rapidly placed CAR BCMA on the spotlight of new therapies being developed for the treatment of this disease. The first clinical study, using a 2nd generation CAR with CD28 co-stimulation, described CRs in several patients and no toxicities associated with off-target effects on other tissues. This dose-escalating clinical trial also determined that a much higher dose (about 10 times more CARTBCMA than CART19) was required to eliminate the tumor. At a 9 × 10^6^ CAR T cell/kg dose, the OR rate was 81% and the median event-free survival was 31 weeks [56,57]. Since then, several centers have put their efforts in developing new CAR BCMA constructs and moving them into clinic. Preliminary results with a 2nd generation CAR using 4-1BB co-stimulation (bb2121 CAR) by Bluebird and Celgene, announced OR of 100%, at a dose of 15–80 × 10^7^ CAR T cells or more [58]. Also LCAR-B38M CAR-T from Nanjing Legend Biotech, which has the particularity of being based on a bi-specific antibody that binds BCMA through two different epitopes, has reported 100% OR and 74% CR [59]. Finally, Novartis has also presented preliminary results of their clinical trial using their CART-BCMA, with 5/6 patients responding at 10^8^ cell dose [60]. Lastly, a new CAR BCMA with successful preclinical results in a mice model (unpublished data) has been developed at our institution (Hospital Clinic, Barcelona). A new Phase I clinical Trial will start in 2019 using this CAR.

**Figure 1:**
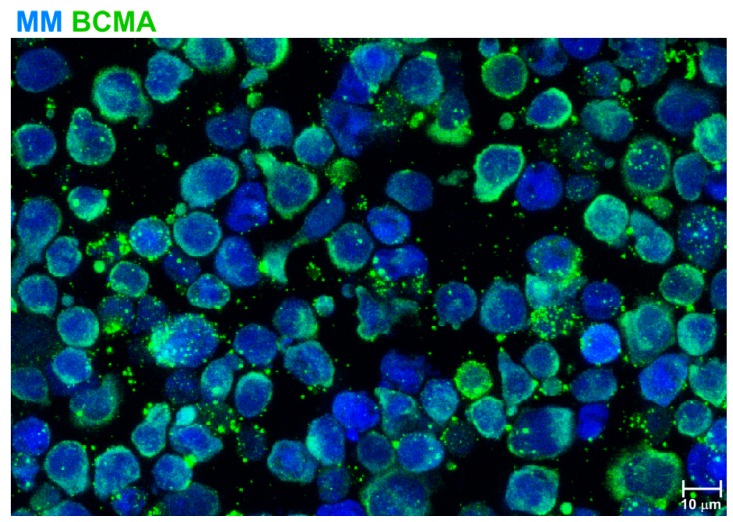
BCMA expression and release from multiple myeloma (MM) cells: MM cells (in blue) stained for BCMA (green). BCMA can be released in vesicles, and shows a non-uniform expression among all MM cells which changes over time. Scale bar is indicated.

Thus, preliminary results using CAR BCMA in the clinic demonstrate that CARs targeting other antigens than CD19 are effective against hematological malignancies. In fact, CAR BCMA and CAR19 seem to be comparable in terms of efficacy and safety, for the treatment of their respective indications. In terms of safety, adverse effects observed with CAR BCMA include CRS in about 75% of patients (10–25% of patients presenting grade 3–4 CRS) and 10% or less of patients presenting neurotoxicity. In all cases, CRS or neurotoxicity resolved, spontaneously or following tocilizumab administration [56,57,58,59,60]. An additional problem which has been observed after CAR BCMA, is the loss of expression of the target antigen [121,123], which has been observed also after CAR 19 treatment [124]. In this sense, we have observed that BCMA expression in MM cells is not stable and it shows a continuous release and recycling into the extracellular milieu (Figure 1). As a possible option to avoid it, the use of γ -secretase inhibitors will avoid the effect of γ-secretase which directly cleaves BCMA and releases soluble BCMA [125].

Several other CARs targeting BCMA have been developed at the pre-clinical level. One that deserves especial mentioning is a CAR based on the natural BCMA ligand, APRIL [61]. APRIL is a proliferating ligand specific for two receptors expressed specifically in MM cells, BCMA and TACI. TACI expression is not as broad as BCMA, but is expressed in 78% of primary MM cells. Pre-clinical data show that APRIL-CAR is as potent as BCMA-CAR in mediating MM killing and may prevent antigen escape by targeting two different proteins. A new clinical trial (NCT03287804) using APRIL-CAR is currently recruiting patients in Europe.

#### 2.4.2. CAR 19

CD19 is not expressed in the predominant population of most of MM cells. Although still controversial, several groups have reported that CD19 expression is associated with a minor subset of malignant cells with a less differentiated phenotype that may act as cancer stem cells [126]. In an attempt to explore the efficacy of CART19 in the treatment of MM, patients received CART19 therapy after ASCT. However, this therapy did not show very promising results, as the time to relapse was not prolonged in 8/10 patients [62,63]. Moreover, a clinical trial at the First Affiliated Hospital, Soochow University, China (NCT03455972) is currently recruiting patients to test efficacy of a bi-specific CD19/BCMA CAR post-ASCT.

#### 2.4.3. CAR CD138

As previously mentioned, CD138 is highly expressed in MM cells, but also in epithelial tissues (reviewed in [91]). Therefore, CD138 might not be an ideal target due to potential associated toxicity of the treatment. However, a study treating 5 MM patients with CAR CD138 did not report significant toxicities [64], although the efficacy of the treatment was limited, with only stable disease observed in 4 out of 5 patients that eventually progressed [64]. Additional studies are required to assess the potential of CAR CD138 for the treatment of MM. In these lines, another clinical trial using this CAR will shortly start at Lineberger Comprehensive Cancer Center, Chapel Hill, NC (NCT03672318). 

#### 2.4.4. Other CARs Being Developed at the Pre-Clinical Stage

CD38 has been proposed as a candidate target for CAR T cell therapy in MM. CD38 is expressed in MM cells and also in other hematopoietic cells, especially NK cells and monocytes, which might lead to high off-target effects. Surprisingly, monoclonal antibody therapies against CD38 in MM patients showed good efficacy and no toxicity associated to off-target effects [17]. However, in vitro data showed that anti-CD38 CAR T cell therapy might present a higher level of toxicity against normal hematopoietic cells [65]. In order to make anti-CD38 CAR T cell therapy safer, several strategies have been proposed and developed in pre-clinical studies, such as designing a low-affinity anti-CD38 CAR [65] or incorporating a caspase-9-based suicide gene to eliminate CAR T cells when required [66].

CD44 is a cell surface adhesion protein expressed in hematological and epithelial cells. It is highly expressed in tumor cells and has a critical role in cancer initiating cells [127]. The expression pattern of CD44v6 splicing isoform is more restricted being absent in hematopoietic stem cells (HSCs) and showing lower level of expression in other hematological cells and keratinocytes [128,129]. An anti-CD44v6 CAR has been developed for the treatment of MM and AML. Pre-clinical data show anti-MM and anti-AML activity and no cytotoxic activity against HSC and keratinocytes. However, cytotoxic activity against circulating monocytes was observed. To circumvent the potential off-target effects of this CAR, a suicide mechanism based on caspase-9 was introduced in the CAR T cells. This system was validated in vitro [67].

Hosen and colleagues described the use of integrin β7 as a potential target for MM [68]. This target was identified using an unbiased screen of >10,000 hybridomas against MM tumor cells. Interestingly, the mAb identified (MMG49) recognizes only a cancer-specific conformation of integrin β7. Therefore, even integrin β7 is expressed in non-tumor cells, the CAR developed is highly specific for MM. Pre-clinical data shows efficacy of MMG49 CAR in vitro and in vivo.

Another antigen being explored as a target for anti-MM therapy is CS1. Pre-clinical studies show anti-MM activity of CS1-CAR T cells in mouse xenograft models, with CS1-CAR-T cells alone [69], or in combination with IMIDs [70]. New phase I clinical trials are expected to open shortly to test the potential of these new CARs as anti-MM therapies in the clinic.

## 3. Concluding Remarks

To summarize, the introduction of ASCT, PIs and IMiDs in the treatment of MM patients have improved significantly the outcome of patients with MM. However, there is a proportion of R/R MM patients to these treatments with a poor outcome where novel strategies are required. The revolution of immunotherapy in cancer treatment in the last years is a field which is being incorporated to the treatment of MM. Among the different types of immunotherapy, the introduction of monoclonal antibodies targeting CD38 (daratumumab) and CS1 (elotuzumab) in combination with IMiDs or bortezomib has significantly improved the outcome of patients with R/R MM. Other monoclonal antibodies targeting IL6, CD56, CD138 and CD40 were tested but, unfortunately, did not show any benefit or just limited responses were observed. Moreover, immunocheckpoint inhibitors have not shown a beneficial effect in MM patients, and additionally, high toxicities were detected. In the field of CAR-modified T cells, BCMA has proven to be the star among other CARs tested in MM and very promising clinical results are expected. CAR T cells targeting CD138 have shown limited responses, however studies in patients are still at initial stages. Other CARs still at pre-clinical phases which could show promising clinical results include CARs against CD38, CD44v6, integrin β7 and CS1. It is expected that in the next decade, as new alternatives appear, novel combination of treatments will be tested and hopefully will lead to higher complete remission rates and prolonged survival in patients with MM along with the lowest possible toxicity.

## Figures and Tables

**Table 1:** Main Clinical trials already finished or on-going with the different immunotherapy options in relapsed/refractory multiple myeloma patients.

Therapeutic Agent	Target	Compound	Combination	Development (Status)	Clinical Trial	Reference
**Monoclonal Antibodies**	CD38	Dara	−	FDA approved	NCT00574288 NCT01985126	[17,18,19]
			Bort and Dex	Phase III (Active, not recruiting)	NCT02136134	[20,21]
			Len and Dex	Phase III (Active, not recruiting)	NCT02076009	[22,23]
	SLAMF7 (CS1)	Elo	−	Phase I (Enrollment halted)	NCT00726869	[24]
			Len and Dex	FDA approved	NCT01393964 NCT00742560 NCT01239797	[25,26,27,28]
	IL6	Siltuximab	Alone or with Dex	Phase II (Completed)	NCT00402181	[29]
			Bort, melpahalan and prednisone	Phase II (Completed)	NCT00911859	[30]
	CD40	Lucatumumab	−	Phase I (Completed)	NCT00231166	[31]
		Dacetuzumab	−	Phase I (Completed)	NCT00079716	[32]
			Len and Dex	Phase I (Completed)	NCT00525447	[33]
	KIRs	IPH2101	−	Phase I (Completed)	NCT00552396	[34]
			Len	Phase I (Completed)	NCT01217203	[35]
	EGFR	Cetuximab	Alone or with Dex	Phase II (Terminated, lack of recruitable patients)	NCT00368121	[36]
	PD-1	Nivolumab	−	Phase I (Recruiting)	NCT01592370	[37]
			Pom and Dex or Elo and Pom and Dex	Phase III (Active, not recruiting)	NCT02726581	
			Elo or Elo, Pom and Dex without Nivolumab	Phase II (Active, not recruiting)	NCT02612779	
			Len	Phase II (recruiting)	NCT03333746	
			Pom and Dex or Elo, Pom and Dex	Phase I (terminated)	NCT03023527	
			Wild-type reovirus, Dex and Carf or Wild-type reovirus, Dex, Carf and Pom	Phase I (recruiting)	NCT03605719	
			Dara or Dara and Cy	Phase II (recruiting)	NCT03184194	
			Alone or Ipilimumab or Lirilumab or Dara, Pom and Dex vs. Dara or Dara	Phase I/II (recruiting)	NCT01592370	
		Pembrolizumab	Pom and Dex	Phase II (Terminated)	NCT02289222	[38]
			Len and low-dose Dex	Phase Ib (Active, not recruiting)	NCT02036502	[39]
			Pom and low-dose Dex	Phase III (Halted)	NCT02576977	[40]
			Len and low-dose Dex	Phase III (Halted)	NCT02579863	[40]
	PDL-1	Durvalumab	Alone or with Pom or Pom and Dex	Phase Ib (Enrollment discontinued)	NCT02616640	
			Dara or Dara, Pom and Dex	Phase II (Enrollment discontinued)	NCT02807454	
		Atezolizumab	Cobimetinib and venetoclax with and without Atezolizumab	Phase Ib/II (recruiting)	NCT03312530	
			Len or Dara or Dara and Len or Dara and Pom	Phase Ib (Recruiting)	NCT02431208	
	TGIT		ASCT	Pre-clinical		[41,42]
**Antibody-Drug Conjugates (ADCs)**	BCMA	GSK285791	−	Phase I (Recruiting)	NCT02064387	[43]
		HDP-1	−	Pre-clinical		[44]
		MEDI2228	−	Pre-clinical		[45]
	CD56	Lorvotuzumab mertansine	−	Phase I (Completed)	NCT00346255	[46]
			Len and Dex	Phase I (Completed)	NCT00991562	[47]
	CD138	BT062	−	Phase I (Completed)	NCT01001442	[48]
			Len and Len / Dex	Pre-clinical		[49]
**BiTEs**	BCMA-CD3	BI 836909	−	Phase I (Recruiting)	NCT02514239	[50]
		EM801		Pre-clinical		[51]
		JNJ-64007957	−	Phase I (Recruiting)	NCT03145181	[52]
		PF-06863135	−	Phase I (Recruiting)	NCT03269136	
	CD138-CD3	STL001	−	Pre-clinical		[53]
**Bi-specific Antibodies**	NKG2D-CS1	CS1-NKG2D biAb	−	Pre-clinical		[54]
	BCMA	BiFab-BCMA		Pre-clinical		[55]
	CS1	BiFab-CS1		Pre-clinical		[55]
**CARs**	BCMA	Anti-BCMA CAR T cells	−	Phase I (Active, not recruiting)	NCT02215967	[56,57]
		bb2121 CAR	−	Phase I (Recruiting)	NCT02658929	[58]
		LCAR-B38M CAR-T	−	Phase I/II (Enrolling by invitation)	NCT03090659	[59]
		CART-BCMA	−	Phase I (Active, not recruiting)	NCT02546167	[60]
	BCMA and TACI	APRIL-CAR	−	Phase I (Recruiting)	NCT03287804	[61]
	CD19	CTL019	ASCT	Phase I (Completed)	NCT02135406	[62,63]
	CD19/BCMA	Bispecific CD19/BCMA CAR	ASCT	Phase I/II (Recruiting)	NCT03455972	
	CD138	CART138	−	Phase I/II (Unknown)	NCT01886976	[64]
		ATLCAR.CD138 Cells	−	Phase I (Recruiting)	NCT03672318	
	CD38	anti-CD38 CAR	−	Pre-clinical		[65,66]
	CD44v6	Anti- CD44v6 CAR	−	Pre-clinical		[67]
	Integrin β7	MMG49 CAR	−	Pre-clinical		[68]
	CS1	CS1-CAR T cells	−	Pre-clinical		[69,70]

Bort: Bortezomib, Dex: dexamethasone, Len: Lenalidomide, Pom: Pomalidomide, Elo: Elotuzumab, Carf: Carfilzomib, Dara: Daratumumab, Cy: Cyclophosphamide, ASCT: Autologous stem cell Transplantation, FDA: Food and Drug Administration.
